# From drug response profiling to target addiction scoring in cancer cell models

**DOI:** 10.1242/dmm.021105

**Published:** 2015-10-01

**Authors:** Bhagwan Yadav, Peddinti Gopalacharyulu, Tea Pemovska, Suleiman A. Khan, Agnieszka Szwajda, Jing Tang, Krister Wennerberg, Tero Aittokallio

**Affiliations:** Institute for Molecular Medicine Finland (FIMM), Nordic EMBL Partnership for Molecular Medicine, University of Helsinki, FI-00014 Helsinki, Finland

**Keywords:** Cell-based drug sensitivity profiling, Drug-target addiction scoring, Experimental-computational target deconvolution, Pan-cancer modeling, Personalized cancer medicine

## Abstract

Deconvoluting the molecular target signals behind observed drug response phenotypes is an important part of phenotype-based drug discovery and repurposing efforts. We demonstrate here how our network-based deconvolution approach, named target addiction score (TAS), provides insights into the functional importance of druggable protein targets in cell-based drug sensitivity testing experiments. Using cancer cell line profiling data sets, we constructed a functional classification across 107 cancer cell models, based on their common and unique target addiction signatures. The pan-cancer addiction correlations could not be explained by the tissue of origin, and only correlated in part with molecular and genomic signatures of the heterogeneous cancer cells. The TAS-based cancer cell classification was also shown to be robust to drug response data resampling, as well as predictive of the transcriptomic patterns in an independent set of cancer cells that shared similar addiction signatures with the 107 cancers. The critical protein targets identified by the integrated approach were also shown to have clinically relevant mutation frequencies in patients with various cancer subtypes, including not only well-established pan-cancer genes, such as *PTEN* tumor suppressor, but also a number of targets that are less frequently mutated in specific cancer types, including ABL1 oncoprotein in acute myeloid leukemia. An application to leukemia patient primary cell models demonstrated how the target deconvolution approach offers functional insights into patient-specific addiction patterns, such as those indicative of their receptor-type tyrosine-protein kinase FLT3 internal tandem duplication (*FLT3-*ITD) status and co-addiction partners, which may lead to clinically actionable, personalized drug treatment developments. To promote its application to the future drug testing studies, we have made available an open-source implementation of the TAS calculation in the form of a stand-alone R package.

## Abstract (precis)

**Drug Discovery Collection:** The authors take a pan-cancer approach that provides functional insights into molecular vulnerabilities across various cancer subtypes and patient-specific target addiction patterns that could lead to actionable treatment strategies.

## INTRODUCTION

With the emerging challenges of translating the cancer genome sequencing information into clinically actionable, targeted drug treatment strategies, it has become evident that we also need complementary strategies for functional investigation of druggable vulnerabilities and cellular addictions of cancer cells. Advances in cell-based high-throughput compound screening have made it possible to profile the cellular activity of an extensive collection of bioactive small molecules, thereby enabling a functional phenotype-based approach to unbiased identification of candidate compounds for individual cancer patients (Pemovska et al., 2013, 2015; Tyner et al., 2013). In addition, such functional information can be used to identify pathway dependencies behind drug sensitivity and resistance patterns in hundreds of cancer cell models across various cancer subtypes (Barretina et al., 2012; Garnett et al., 2012; Heiser et al., 2012; Basu et al., 2013). Compared with transcriptomic-based functional classification of cancer cells, which predominantly reflects their tissue of origin (Klijn et al., 2015), comprehensive drug sensitivity profiling provides more actionable information in the preclinical setting, which may be used subsequently to guide the development of personalized treatment regimens or to design so-called basket clinical trials (Redig and Janne, 2015).

Identification of the cellular targets behind the observed drug response profiles (so-called target deconvolution) is an important aspect of the rational phenotype-based drug discovery approach (Terstappen et al., 2007). Knowledge of the active target sub-space of those drug compounds that show potency in the cell models is fundamental for understanding their mechanism of action, which in turn is critical for the drug development process as well as for drug repurposing applications. Systematic mapping of the full activity spectrum, ranging from the compound's primary targets to its secondary or ‘off-targets’, provides a basis for the development of more effective and safe therapeutic options that could also avoid severe side effects. Such system-wide addiction maps may offer insights not only into the mechanism of action of the compounds, but also means for improved understanding of their cell type-specific and cancer-selective efficacies. There is a broad array of experimental strategies for phenotype-based target deconvolution (Terstappen et al., 2007). Computational target deconvolution can guide the purely experimental approaches, but the existing computational approaches have focused on a single cancer type only or are limited merely to kinase inhibitors (Tyner et al., 2013; Yadav et al., 2014; Szwajda et al., 2015).

In the present study, we applied an experimental-computational target deconvolution approach in a diverse collection of cancer cell models. Our computational target addiction scoring (TAS) algorithm integrates cell-based compound sensitivity profiling with global drug-target interaction networks that capture a broad spectrum of both on- and off-target effects of the compound panel, comprising not only kinase inhibitors but also other important drug target classes. The integrated approach is applicable both to cell line models *in vitro* and to patient-derived samples *ex vivo*; for a given cancer cell model, it provides a functional importance scoring of the drug targets, according to an estimate of how much the cell sample is addicted to the particular target protein. In an application to >100 cell lines, we deconvoluted the target signal patterns across the heterogeneous cancer cell collection, hence revealing pan-cancer correlations in their addiction signatures between both shared and unique genetic and tissue backgrounds. A specific application to leukemia patient primary cancer cell models demonstrates how the target deconvolution approach can yield functional and actionable insights into patient-specific addiction patterns towards clinical translation.

**RESOURCE IMPACT**

**Background**

Cell-based drug sensitivity testing is becoming a popular approach to provide a functional profile not only for cancer cell lines but also for primary patient cells to map pathway dependencies in specific cancer subtypes and to select optimal personalized treatments for cancer patients. However, several critical challenges remain; in particular, how to deconvolute signals of addiction – a phenomenon describing the dependency of certain tumors on a single oncogene for growth and survival – behind the drug sensitivity and resistance phenotypes, which is a critical step in the phenotype-based drug development process and an important prerequisite for many translational applications.

**Results**

In this study, the authors implemented a network-based experimental-computational approach that makes use of polypharmacological effects of drug compounds to identify pharmacologically actionable target signals in individual cancer cell subtypes and in patient-derived cell models. When applied to >100 cancer cell lines, this approach mapped target signal profiles across heterogeneous cancer cell models; these profiles revealed pan-cancer correlations in the addiction signatures of different cancer cell subtypes that have both shared and unique genetic and tissue backgrounds. Specific application to leukemia patient primary cell models demonstrated how the target deconvolution approach offers functional insights into patient-specific addiction patterns that might lead to clinically actionable treatment strategies.

**Implications and future directions**

This computational target deconvolution method represents an open-source and extendable approach that can be used to identify target addiction profiles across heterogeneous cancer cell lines and leukemia patient primary cell models. This approach should thus provide a valuable resource for the community to enable translation of drug sensitivity testing results into many exciting drug development and repurposing opportunities.

## RESULTS

### Target addiction scoring in cell-based models

**Fig. 1.**
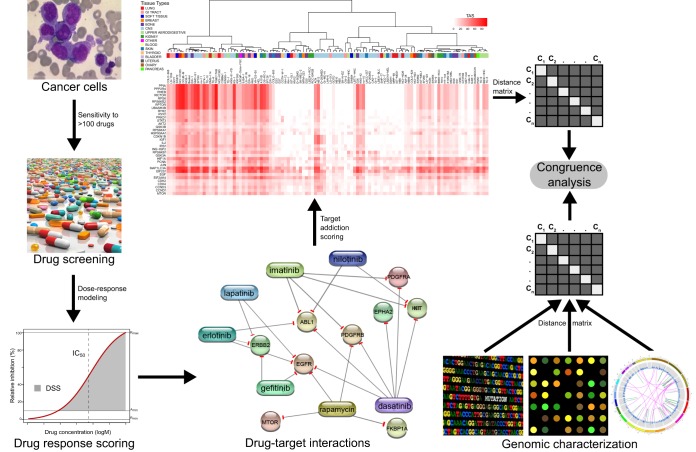
**Schematic diagram of the target addiction scoring.** The approach uses as its input the response profiles of a cancer sample to a large panel of bioactive compounds, and then transforms this response profile into a target addiction score using information encoded in system-wide drug-target networks. The example network was constructed using STITCH, but other drug-target sources were also used here (see Materials and Methods for details). Only a sub-set of the 749 protein targets of the full target addiction score (TAS) profiles is illustrated here as calculated across the 107 cancer cell lines (the full target addiction matrix is available in supplementary material Table S1). As the drug response metric, here we used the drug sensitivity score (DSS), which combines multiple dose-response parameters, including the standard half-maximal inhibitory concentration (IC_50_), in the area under curve (AUC) calculation. DSS differs from standard AUC in terms of using both the maximal asymptotic response (*R*_max_) and the minimal activity threshold (*A*_min_) when normalizing the response metric (Yadav et al., 2014). The target addiction signatures of the cancer cells were compared with those from the genomic signatures of the same cell lines using distance-based congruence analysis.

We implemented an experimental-computational platform that identifies critical drug targets in individual cancer cell lines or patient-derived cell models (Fig. 1). The approach takes as its input the observed response profile of a particular sample to a large and broad panel of bioactive compounds (Pemovska et al., 2013), and then transforms this phenotypic profile into a target addiction profile, through information encoded in system-wide drug target networks that connects the compounds to their wide classes of cellular targets (see Materials and Methods for the details of the collection of target information for the compound panels used in this study). Systematic mapping of such target addiction signatures provides a ranking of the drug targets according to their functional importance in the given cancer sample. The network pharmacology approach aims to identify druggable signal addictions, that is, such molecular vulnerabilities of a given cancer sample that are pharmacologically actionable and may therefore lead to straightforward drug development or repurposing opportunities. Here, we used primary leukemic cells as an example disease model to demonstrate how this target deconvolution approach may lead to novel therapeutic developments in patient-derived cell models.

We first systematically tested the TAS approach using the comprehensive cell line data from the Genomics of Drug Sensitivity in Cancer (GDSC) project, which is the largest public resource for information on drug sensitivity in cancer cells along with their comprehensive genomic and molecular characteristics (Yang et al., 2013). We calculated the TAS profiles for the subset of 107 cell lines with complete drug responses to the full panel of 138 drug compounds under clinical and preclinical investigation (supplementary material Table S1). As the primary drug response metric, we used the drug sensitivity score (DSS), which was recently shown to improve the prediction of drugs' mechanism of action, as well as the identification of drug-responsive cell lines and patient-derived cancer cells (Yadav et al., 2014; Pemovska et al., 2015). We also tested the performance of other response metrics, including the standard half-maximal inhibitory concentration (IC_50_), as well as the area under the dose-response curve (AUC), as available in the GDSC resource. The pairwise addiction signatures between the cell lines were compared with those derived from the genomic signatures of the selected cell lines, using congruence analysis, and later the predictive power of the TAS signatures was assessed in an additional set of 20 cell lines from GDSC.

### Comparison with tissue type and genomic profiles

**Fig. 2.**
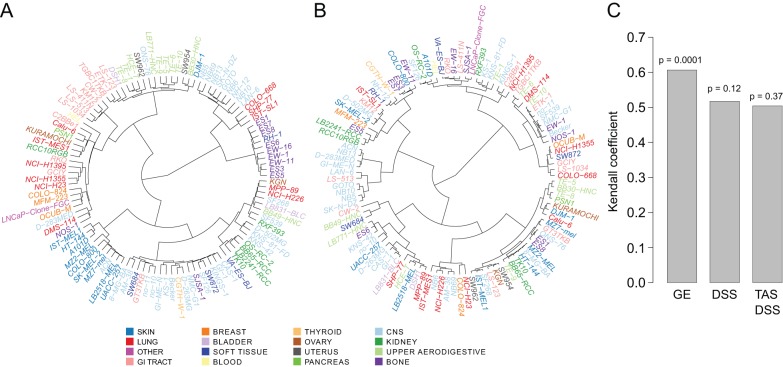
**Functional classification of the 107 cancer cell lines.** (A) Clustering based on genome-wide transcriptomic profiles. (B) Clustering based on target addiction profiles. Color coding indicates the tissue of origin. (C) Comparison of the pairwise distances between the cell lines calculated using either their tissue type (binary distance matrix) or the gene expression (GE) and DSS profiles, with and without using the TAS transformation (cosangle distance matrix). Statistical significance was assessed by permutation-based testing of Kendall's correlation coefficient.

We first derived unsupervised molecular and functional classifications of the 107 cancer cell lines based on their transcriptional, drug response and target addiction profiles. As expected, the cancer cell line clustering based on the gene expression profiles was largely driven by the tissue origin of these cells (Fig. 2A,C; *P*=0.0001, permutation test of the Kendall coefficient). In contrast, the cell line clustering based on the target addiction profiles was effectively independent of the tissue type (Fig. 2B; supplementary material Fig. S1). The same was also true when using the DSS alone, although not as prominent as with the DSS-TAS transformation (Fig. 2C; supplementary material Fig. S2). For instance, gastrointestinal (GI) tract cell lines formed a clear sub-cluster in the expression-based clustering, whereas these cells were scattered in various sub-clusters of the TAS-based functional classification. This indicates that the pan-cancer functional relationships in the addiction patterns carry information beyond the tissue origin alone, which may therefore be more closely connected to the underlying biology shared by multiple cancer subtypes.

**Fig. 3.**
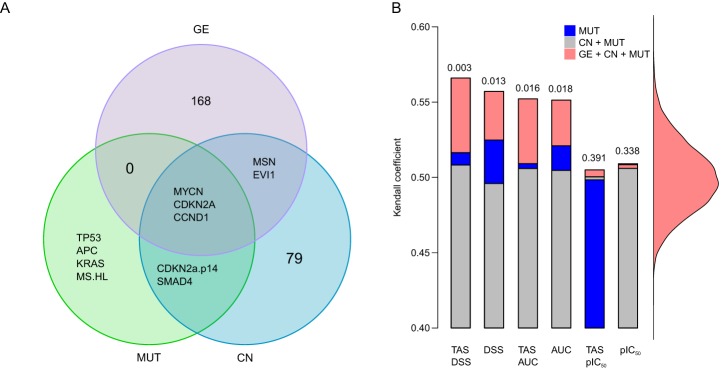
**Comparison of addiction profiles with genomic profiles.** (A) Overlap of genes in active components shared by gene expression (GE), copy number (CN) and mutation status (MUT) in the Bayesian group factor analysis (GFA). (B) Comparison of the pairwise cosangle distances between the cell lines calculated based on their genomic signatures or the TAS, with different drug response metrics (DSS, AUC and IC_50_). Color coding indicates the different genomic data sets used in the congruence analysis. Statistical significance is marked at top of the combined genomic bar, assessed by its permutation-based background distribution of the Kendall coefficient (right).

To determine the degree to which the target addiction-based classification of the cancer cells could be explained by their pan-cancer genomic or molecular correlates, we next compared the similarities and differences in the TAS patterns with those derived from the genomic and molecular signatures of the same set of 107 cell lines. In order to capture effectively the joint variability between gene expression (GE), copy number (CN) and mutation status (MUT) across the genetically heterogeneous cancer cells, we used a sparse extension of the Bayesian group factor analysis (GFA; Virtanen et al., 2012; Khan et al., 2014), which enabled us to identify a shared set of active components between these three high-dimensional genomic and molecular data sets (supplementary material Fig. S3B). The novel GFA analysis resulted in both unique and common genes in the shared active components, including, for instance, well-known cancer oncogenes, such as *MYCN* and *CDKN2A*, which were common to all the three data sets, whereas *TP53* and *KRAS* were unique to the MUT data set only (Fig. 3A).

When comparing the target addiction signatures and the combined genomic signatures from the GFA analyses, it was found that the TAS version calculated with DSS led to the overall best concordance (Fig. 3B; *P*=0.003, permutation test). Likewise, the AUC response metric showed slightly improved concordance after its TAS transformation, whereas the IC_50_ metric showed a totally opposite trend in concordance. Surprisingly, the combination of the CN and MUT components resulted in a lower concordance with the addiction signatures, compared with that when using the mutation status alone, perhaps owing to the relatively high dependency (anti-correlation) of the genomic information captured by the copy number alterations and somatic point mutations, as observed in recent pan-cancer analyses (Ciriello et al., 2013). These results were based on systematic concordance analysis using various distance measures on both functional and genomic profiles, which supported the overall good performance of the cosangle dissimilarity (otherwise known as uncentered correlation; supplementary material Fig. S4).

### Robustness and predictive power of addiction signatures

**Fig. 4.**
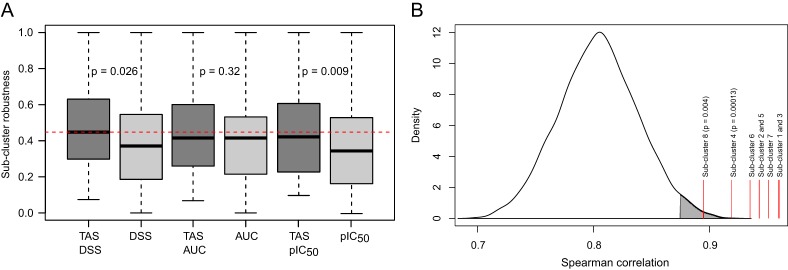
**Robustness and predictive power of addiction profiles.** (A) Robustness of the sub-clusters based on 10,000 bootstrapped samples. The red dotted line indicates the median robustness of the TAS-DSS combination (supplementary material Figs S5 and S6 show the robustness values separately for each individual sub-cluster in the clustering dendrogram). Statistical significance of the difference in robustness with and without TAS transformation was assessed using the Wilcoxon rank-sum test. (B) Background gene expression correlation among all the cancer cell line pairs. The red continuous lines indicate the average expression correlation of the new sets of cell lines with those predicted to share a similar addiction pattern based on the TAS clustering (supplementary material Fig. S7 shows the eight detected sub-clusters). Empirical statistical significance was assessed using the background null distribution.

To verify that the TAS-based cell line cluster solutions were not obtained by chance, for instance, owing to the specific data sample only or a single run of the clustering algorithm, we repeated the cell line clustering on 10,000 bootstrap samples of the original 107 cell lines. Strikingly, the robustness of the cell line classifications based on each drug response metric could be improved by using the TAS transformation (Fig. 4A), most significantly with the IC_50_ metric (*P*=0.009, Wilcoxon rank-sum test). Although the average robustness values over the whole spectrum of sub-cluster structures were relatively similar between the three response metrics, the DSS-TAS combination resulted in the most consistent cluster solution over the bootstrap samples. Notably, each of the cluster solutions made without the TAS transformation had sub-clusters with 0% robustness also at the upper levels of hierarchy in the corresponding dendrograms (supplementary material Fig. S5), indicating that these larger sub-clusters at higher levels were in fact very sensitive even to small changes in the drug response input data. In contrast, the TAS-based cluster solutions resulted in notably higher robustness values for each sub-cluster, especially at the upper levels of the dendrogram (supplementary material Fig. S6), compared with their TAS-free counterparts (supplementary material Fig. S5).

To evaluate whether the DSS-TAS profiles could also carry a predictive signal for the genomic characteristics of the cancer cell lines, we combined 20 additional cell lines from GDSC (tested with fewer than 138 compounds) with the original analyses done on the basis of the 107 cell lines (supplementary material Table S1). Within each of the detected eight addiction sub-clusters, we compared the GE, CN and MUT profiles of the newly added cell lines with those of the original cell lines, often representing different tissue types (supplementary material Fig. S7). As expected, the gene expression profiles were highly correlated among all the cancer cell line pairs, leading to a shifted background distribution with a positive mean (Spearman correlation 0.8). However, the expression correlations of the new cell lines with those predicted to share similar addiction patterns were significantly higher (Fig. 4B; *P*<0.004, empirical significance based on the permuted null distribution). Likewise, the TAS-based clusters were fairly predictive of the CN profiles, whereas the mutation status of the new cell lines could not be predicted accurately (data not shown). This is likely to result from the relatively small number of genes whose mutation status was available (out of the 84 genes profiled in GDSC, only 51 genes harbored mutations in these 107 cell lines).

### Clinical relevance of the identified addiction signatures

**Fig. 5.**
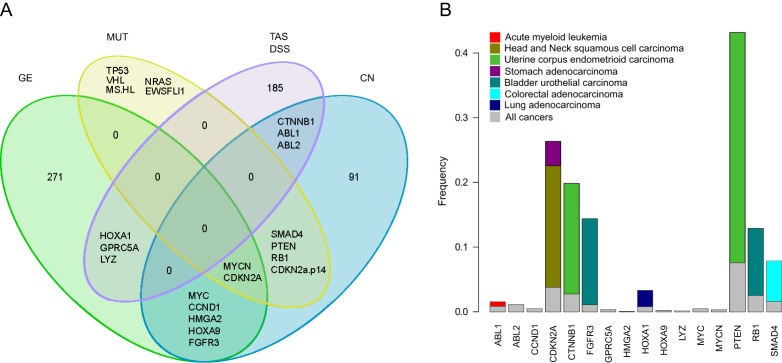
**Clinical relevance of the identified addiction signatures.** (A) Overlap of genes in active components shared by GE, CN, MUT and the TAS-DSS in the GFA. (B) Frequency of the mutations in cancer patients from The Cancer Genome Atlas (TCGA). Color coding indicates different cancer subtypes selected based on their frequency distribution being different from that of all the cancers. Each bar plot for the cancer subtypes starts from zero.

Next, we combined the target addiction signatures of the cancer cell lines into GFA in order to study the similarities and differences in the active components revealed by the genomic and functional profiles. Overall, the shared active components had only a few common genes, indicating the complementary nature of these profiles (Fig. 5A). In particular, the DSS-TAS active component shared only three genes with both the GE (*HOXA1*, *GPRC5A* and *LYZ*) and CN (*ABL1*, *ABL2* and *CTNNB1*) and none with the mutational profiles; although this surprisingly small overlap may be attributed in part to the rather sparse mutation panel, it also demonstrates that the functional drug/target profiling provides added value to the genomics-only-based analyses and that the targets deemed functionally critical by the addiction scoring are not necessarily overlapping with those detected by genomic analyses of the same cancer cells.

As a specific example, we focused more closely on the four common genes that were included in the shared active components between DSS-TAS, GE and CN (*ABL1*, *ABL2*, *GPRC5A* and *CTNNB1*). An interaction network was constructed by combining these genes with each other using direct, experimentally validated and manually curated interactions from the Ingenuity Pathway Analysis knowledgebase (IPA; QIAGEN, www.qiagen.com/ingenuity). In addition to novel links, the network analysis also identified missing players between these four genes, including *RAS* oncogene, *EGFR* and *BCR*, all of which are well established as having a role in the pathogenesis and treatment of many cancer types, including breast, colorectal, pancreatic and lung cancer; these molecules are either serving as biomarkers for cancer prognosis and/or prediction of treatment efficacy (supplementary material Fig. S8).

We next investigated how frequently the genes shared by at least two data sets are mutated in cancer patients using data from The Cancer Genome Atlas (TCGA) resource. In addition to pan-cancer recurrent genes, such as *PTEN*, which are mutated at high frequency in multiple cancers, this analysis also revealed a number of genes that are more uniquely mutated in specific cancer types; for instance, *ABL1* in acute myeloid leukemia (AML) and *HOXA1* in lung adenocarcinoma (Fig. 5B). In leukemia, the *BCR-ABL1* fusion gene is a known driver oncogene in chronic myeloid leukemia and Philadelphia chromosome-positive acute lymphoblastic leukemia. Using an unbiased drug sensitivity testing of primary leukemia cells *ex vivo*, we recently identified the tyrosine kinase inhibitor axitinib as a selective and highly effective inhibitor for *T315I*-mutant *BCR-ABL1*-driven leukemia (Pemovska et al., 2015).

### Application of TAS to primary leukemia patient cells

Using leukemia as an example disease model, we carried out an ‘*in silico* personalized medicine’ case study. Specifically, we applied the TAS approach to the cell-based drug response data obtained from a recent study where the sensitivity of primary leukemia patient cell samples was assessed to a panel of 66 kinase inhibitors *ex vivo* (Tyner et al., 2013). The TAS signatures across the 151 leukemia samples revealed patient-specific addiction patterns that were not explained by their diagnostic classification (supplementary material Table S2). Based on our previous analysis of *ex vivo* drug response patterns in AML patients, where receptor-type tyrosine-protein kinase FLT3 internal tandem duplication (*FLT3-*ITD) mutation status was found to be associated with functional classification of the AML subtypes (Pemovska et al., 2013), here we focused specifically on the *FTL3-*ITD addiction levels across the leukemia patient samples.

**Fig. 6.**
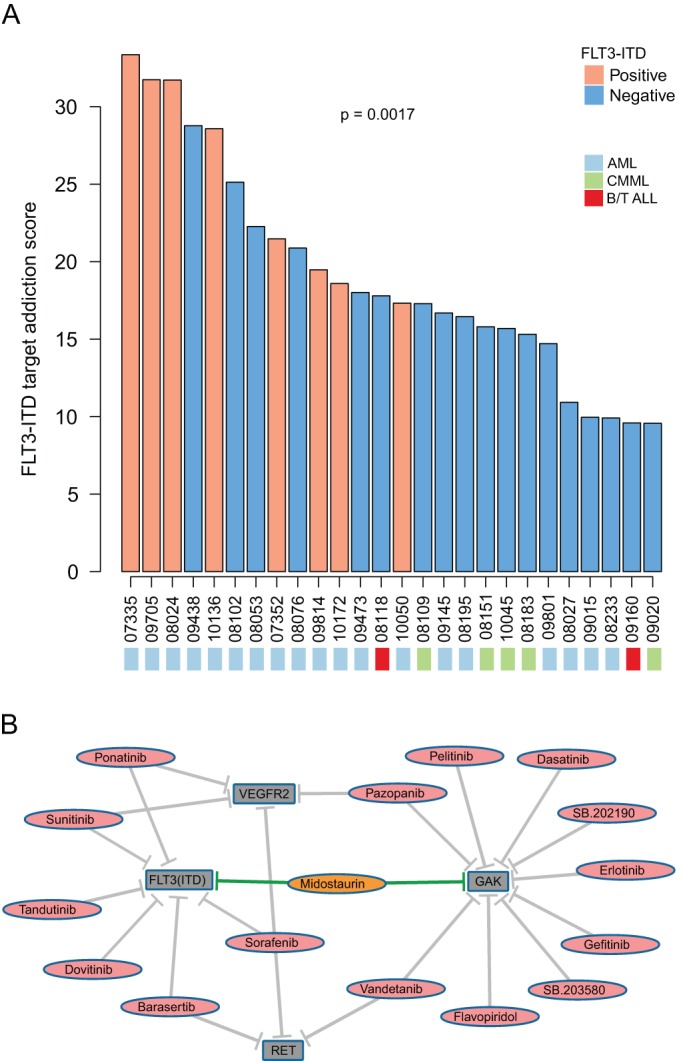
**Application of the addiction scoring to leukemia patients.** (A) Ranking of the leukemia patients based on their addiction to *FLT3-*ITD. Only a subset of those patients is shown with known *FLT3-*ITD status as determined with standard molecular diagnostic testing in the original study (Tyner et al., 2013). Statistical significance of the distinction between the positive and negative *FLT3-*ITD cases was assessed using the Wilcoxon rank-sum test. Color coding indicates the patient diagnostic subtypes (more detailed sample characteristics are listed in supplementary material Table S2). (B) Compound-target interaction network combining the four selected target kinases (gray nodes) with their unique and shared inhibitors (red nodes). For illustrative purposes, only the most potent compound-target interactions were included based on the target classification from Tyner et al. (2013).

In the sub-set of the leukemia patients with known *FLT3-*ITD status, the TAS-based ranking distinguished the positive and negative cases (Fig. 6A; *P*=0.00017, Wilcoxon rank-sum test). PCR analysis revealed one of the patient samples (ID 08024) to exhibit *FLT3-*ITD with loss of the wild-type *FLT3* allele in the original study (Tyner et al., 2013); in contrast, our target addiction analysis revealed a number of additional patient samples having strong addiction to *FLT3-*ITD, and also some without this mutation detected in the standard molecular diagnostic test (e.g. ID 09438). The calculation was based on a number of compounds with both on- and off-targeting of *FLT3-*ITD, including sunitinib, sorafenib and ponatinib (Fig. 6B), all of which are approved for other indications, and may therefore lead to straightforward drug repurposing opportunities.

To investigate whether the target addiction scoring can also reveal co-addiction relationships between drug targets, we correlated the addiction profile of *FLT3-*ITD with all of the other kinase targets across the 151 leukemia patient samples. This led to the identification of significant *FLT3-*ITD co-addiction partners *VEGFR2*, *RET* and *GAK* (Spearman correlation >0.94, Bonferroni-corrected *P*<10^−5^). Whereas *VEGFR2* and *RET* shared a number of common inhibitors with *FLT3-*ITD, partly explaining their correlated addiction profiles, *GAK* displayed a more distinct inhibitor panel, with only one potent inhibitor common with *FLT3-*ITD (midostaurin; Fig. 6B). Such distinct addiction correlations may imply multitargeting opportunities for treating *FLT3-*ITD-driven leukemia, which currently lacks effective targeted treatment options.

## DISCUSSION

We have shown in the pan-cancer cell-based approach that the target addiction profiles provide functional information that is effectively independent of the tissue of origin, hence making it possible to start capturing functional relationships in molecular addiction patterns across various cancer subtypes. This is in line with recent pan-cancer analyses that have found shared mutation patterns behind many otherwise unrelated cancer subtypes (Alexandrov et al., 2013; Ciriello et al., 2013; Kandoth et al., 2013; Hoadley et al., 2014; Leiserson et al., 2014). However, we found that the information encoded in the addiction signatures was only concordant in part with that extracted from their genomic signatures, suggesting that similarities observed in the addiction patterns between cancer cells cannot be attributed solely to their shared genetic background. This demonstrates that the functional target addiction profiling provides complementary information, when compared with the genomics-only-based profiling of cancer cells, which has not yet fulfilled its promises in providing clinically actionable therapeutic strategies for many cancer types. In contrast, addiction signatures provide pharmaceutically actionable insights into the importance of druggable protein targets in a cell-type-specific manner, which could support the development of personalized therapeutic strategies for many cancer types. However, additional levels of genomic and molecular information should be included in future studies, including chromosome re-arrangements, protein activities and methylation changes, to study in more detail the genomic and molecular correlates behind the observed addiction patterns.

Genomics-based analyses typically identify cancer-related genes as those that are frequently mutated in large cancer cohorts. Such cancer drivers are often classified into either loss of function (e.g. tumor-suppressor genes whose perturbation contributes to tumorigenesis) or gain of function (e.g. oncogenes whose abnormal activity provides a growth advantage to the cancer cells). In practice, only proteins with activating mutations can be targeted directly with small-molecule inhibitors. In contrast, loss-of-function cancer genes, including *TP53* or *PTEN*, could be targeted through other means, such as those based on the concept of synthetic lethality (Rubio-Perez et al., 2015). However, beyond a few successful examples, these strategies are not yet very mature in clinical practice. Therefore, oncoproteins such as *ABL1*, which have been found to be mutated only in a subset of leukemia patients, could be more directly actionable with *ABL1*-targeting tyrosine kinase inhibitors, such as imatinib, nilotinib, dasatinib, bosutinib and ponatinib, all of which are approved for *ABL1*-driven diseases. Likewise, *HOXA1*, found to be mutated in lung adenocarcinoma, is associated with a hypermethylation phenotype (Tsou et al., 2007; Selamat et al., 2011); hence, it might be targetable by DNA methyltransferase inhibitors, such as decitabine and azacitidine, suggesting a molecularly stratified treatment strategy for this patient population.

In the leukemia patient application, we focused specifically on the activating internal tandem duplication (ITD) mutations in *FLT3* (*FLT3-*ITD), which are detected in approximately 20% of AML patients associated with a poor prognosis (Thiede et al., 2002). Clinical trials have not yet been able to demonstrate a significant clinical benefit for *FLT3-*ITD inhibition monotherapies, suggesting significant rewiring and cross-play within and between multiple signaling pathways during the disease development and treatment relapse. Therefore, our results showing correlated addiction patterns between *FLT3-*ITD and other targets, such as *GAK*, may provide leads for more effective combinatorial targeting of *FLT3-*ITD-driven diseases. It is known that *GAK* plays a crucial role in clathrin-mediated membrane trafficking and maintenance of centrosome maturation and mitotic chromosome assembly (Eisenberg and Greene, 2007; Sato et al., 2009; Shimizu et al., 2009). In addition, *GAK* is critical for cell growth (Lee et al., 2008), highly expressed in different cancer cell types, and has been shown to regulate the epidermal growth factor receptor (EGFR; Zhang et al., 2004), as well as to serve as a transcriptional coactivator of androgen receptor (Ray et al., 2006). *GAK* is also involved in regulation of hepatitis C viral entry and assembly (Neveu et al., 2015), and pathogenesis of Parkinson's disease (Dumitriu et al., 2011). Given such wide implications in cellular processes, *GAK* might represent a critical node to target along with more established targets to achieve clinical benefit in *FLT3-*ITD patients, provided this leads to a tolerable toxicity profile.

To model the polypharmacological effects and mechanism of action of multitargeting compounds, we wanted to consider here a wide spectrum of both direct and indirect compound-target interactions (supplementary material Table S3), because the observed drug response (that we want to capture) is elicited not only through the direct binding targets, but also through secondary and downstream effects (Xie et al., 2012). Therefore, we extended the compound-target mappings beyond the primary targets listed in the GDSC (Yang et al., 2013) by collecting both on- and off-targets for the 138 compounds using other publicly available resources. For the kinase inhibitors, we extracted bioactivity data from large-scale selectivity profiling assays, including those by Davis et al. (2011) and Metz et al. (2011), which have been shown to provide high-quality, quantitative bioactivity data (Tang et al., 2014). Compared with the existing computational target deconvolution methods, we also extended the drug target mapping beyond the kinase targets only. For the non-kinase targeting compounds, we used data available in drug databases, such as ChEMBL17 (Gaulton et al., 2012) and STITCH4 (Kuhn et al., 2014). In particular, STITCH provided a relatively extensive list of additional target annotations for many of the compounds, not available from the other resources (supplementary material Fig. S9). Even though we made our best effort to include only the most reliable targets, through stringent filtering criteria and manual curation, it is likely that the target mappings from these databases include both false-positive and false-negative interactions (Tang et al., 2014). Although the TAS results showed a relatively robust behavior (supplementary material Fig. S10), future improvements in both the compound and target coverage of the quantitative bioactivity mappings should lead to further improvements in the performance of computational target deconvolution methods.

Another challenge with the cell-based drug sensitivity testing is how to distinguish selective responses, i.e. those observed in the specific cancer subtype only, from those observed in many or all of the cancers, which often lead to toxic side effects in normal cells. For distinguishing selective drug responses, it would be beneficial in future cancer cell line screening efforts also to include a panel of ‘normal’ cell types, through which to estimate the degree of side-effect toxicity. This also applies to the studies with patient-derived cell models, where reference drug response profiles from healthy control subjects have been helpful to sort out the cancer-selective responses (Pemovska et al., 2013). In large-scale efforts, such as GDSC or Cancer Cell Line Encyclopedia (CCLE; Barretina et al., 2012), the pan-cancer reference panel might enable one to estimate the background distribution of the spectrum of drug response patterns generalizable to most cell types. Likewise, the target addiction profiles can be compared across several cancer subtypes, or against controls when available, to estimate the cancer-specific or subtype-selective addiction patterns. Furthermore, in clinical applications, the differential target addiction profiles across the disease evolution and relapse should provide important insights into mechanisms behind treatment sensitivity and resistance, and potentially even suggestions for the second-line treatment alternatives for the relapsed patients.

We have previously shown in a targeted application to a subset of breast cancer cell lines, which were perturbed with 40 kinase inhibitors, that such differential addiction scoring can extract important information from heterogeneous triple-negative breast cancer cells toward designing multitargeted combinatorial strategies that show synergistic inhibition effects (Szwajda et al., 2015). The present work extends this concept to a pan-cancer approach, using a wide collection of 107 cancer cell line models of various tissue origin, as well as broad panel of 138 compounds, including not only kinase inhibitors but also other important drug target classes. Importantly, a specific case study in leukemia patient primary cells demonstrated how this concept is also applicable to patient-derived cancer cell models. There are multiple ways in which the current computational pipeline could be improved; for instance, using probabilistic models that allow multilabel soft clustering and also incorporating functional annotation of genes to guide the clustering procedure and to take into account the multitarget effects of many compounds (Flaherty et al., 2005); however, already the standard hierarchical clustering algorithm was shown to provide surprisingly robust solutions to the data resampling. Strikingly, the shared addiction patterns within the detected cell line sub-clusters were also highly predictive of the transcriptomic patterns among independent sets of additional cancer cells. An open-source and easily extendable implementation of the TAS calculation is made freely available to support its tailored application to translating drug sensitivity testing results into addiction scores, which may lead to many exciting drug development and repurposing applications.

## MATERIALS AND METHODS

### Cancer cell line material

High-throughput drug screening and molecular and genomic profiling data for 639 cancer cell lines were available from Garnett et al. (2012), which included pre-computed area under the dose-response curve (AUC) and half-maximal inhibitory concentration (IC_50_) response parameters. We had access also to the raw dose-response data (kindly provided by Dr Mathew Garnett, Wellcome Trust Sanger Institute, UK), enabling us to compute our drug sensitivity score (DSS; Yadav et al., 2014) for all the compounds and cell lines. Given that not all the cell lines were screened against all the compounds, we initially chose only the subset of 117 cell lines, which were tested with all 138 compounds. This set of cell lines was filtered further based on the availability of genomic and molecular profiles, including gene expression, mutation and copy number profiles. After filtering, we had a total of 107 cell lines with complete drug testing and molecular profiling data, which were used in our further analyses. The IC_50_ response parameter was converted into pIC_50_ by taking −log10 of IC_50_ at molar concentration. The mutation data in Garnett et al. (2012) contain such somatic mutations in 84 genes from COSMIC that affect the protein sequence or function; these were treated as binary genomic profiles in our analyses.

### Drug-target interactions

For the system-level modeling of drugs' mode of action and polypharmacological effects, we extended the compound-target mappings beyond the direct binding interactions and the primary targets listed at Genomics of Drug Sensitivity in Cancer (GDSC) database of the Sanger Institute (http://www.cancerrxgene.org/); more specifically, we also collected secondary and downstream targets for the compounds using a number of additional resources. For the kinase targeting compounds, we extracted quantitative bioactivity data from the large-scale kinase inhibitor selectivity profiling assays by Davis et al. (2011) and Metz et al. (2011). For the non-kinase targeting compounds, we collected target data available in public drug databases, such as ChEMBL17 (Gaulton et al., 2012) and STITCH4 (Kuhn et al., 2014). We selected most reliable targets from STITCH having either experimental score >10 and combined score >500 or experimental score <10 and combined score ≥900. For a selected 24 compounds, we extended their target space with experimental scores ≥300. From the ChEMBL, Davis et al. (2011) and Metz et al. (2011), we selected compound-target links with bioactivity parameters IC_50_, *K*_d_, *K*_i_ and potency ≤35 nM to focus on the most potent targets only. We also manually filtered out genes involved in cellular metabolism, such as cytochrome (CYP) and ATP-binding cassette (ABC) genes. This resulted in an interconnected network among 138 compounds and 749 protein targets, capturing both direct and indirect interactions, which was used for modelling the comprehensive spectrum of not only on-target but also off-target and downstream effects of the compounds (supplementary material Table S3).

### Target addiction scoring

The target addiction score gives an estimate of the sensitivity of a cell to the inhibition of a particular protein target, thereby extending the concept of kinase addiction maps introduced by Pemovska et al. (2013) and Yadav et al. (2014). Formally, given a drug response profile in a specific cell sample, as obtained from response metrics such as DSS, AUC or pIC_50_, the target addiction score (TAS) for a particular target *t* is calculated by averaging the observed drug response (DR) over all those *n_t_* compounds that are known to target protein *t*, as follows:


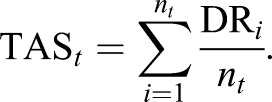
Mathematically, TAS defines a transformation between the spaces spanned by the compounds and their targets, which maps observed drug responses to the underlying target addictions. Calculating TAS for various cancer cell lines provides pan-cancer information about their shared and unique addiction signals. Calculating TAS in patient-derived samples provides insights into the individual-level druggable vulnerabilities. The TAS calculation is available as open-source R implementation at https://bitbucket.org/BhagwanYadav/target-addiction-score-tas-calculation.

### Group factor analysis

Factor analysis is a statistical method, which allows description of the dependencies among many observed variables using a few factors. Group factor analysis (GFA) is a recent extension of the standard factor analysis to capture joint variability across multiple data sets (Virtanen et al., 2012; Khan et al., 2014). Here, GFA was applied to the genomic and molecular data sets, namely gene expression, copy number and mutation profiles. GFA segregates the components that are shared among all the data sets from those shared among some or only one data set. The genomic and molecular profiles comprised gene expression of 13,321 genes, copy number of 426 genes and mutational status of 84 genes. The gene expression was filtered for the most strongly varying genes across the cell lines (s.d. >2), leading to 951 genes. From mutation data, we selected 51 genes such that each gene is mutated in at least one of the 107 cell lines. The gene expression and copy number data were converted to log_2_ scale. Supplementary material Fig. S3A shows the standard deviation of all genes in the expression data. The genes with high enough standard deviation (above the red line) were used in GFA. All the data sets were scaled to unit standard deviation, giving them equal weight. GFA was run with a varying number of components (K) to model the total variation in the three data sets. Supplementary material Fig. S3B shows the activity of the components in GFA. A component is present for a data set when shown in black, and otherwise absent. The run resulted in components absent in all the data sets, indicating that *K*=30 was large enough to model the data.

### Congruence analysis

Kendall's coefficient of concordance (*W*) is a non-parametric statistic to assess the agreement between two rankings (Kendall and Smith, 1939; Legendre, 2005), which ranges from 0 (no agreement) to 1 (complete agreement). Here, it was applied to distance matrices, consisting of pairs of cell lines, based on different data sets, such as drug sensitivity, target addiction, gene expression, copy number, mutation status or their shared components from the GFA. We computed *W* to investigate the congruence between two distance matrices using ‘ape’ R package (Paradis et al., 2004). We systematically evaluated congruence using different distance measures, such as Euclidean, Manhattan, Pearson, Spearman, Kendall and cosangle distance. We found that cosangle was the most significant distance measure in our analysis (supplementary material Fig. S4). The statistical significance of the congruence was assessed by performing 10,000 permutations. We also performed Mantel's randomization test using ‘ade4’ R package (Dray and Dufour, 2007) and obtained similar results to those of Kendall-based congruence analysis. For the congruence analyses with tissue type information, we represented each cell line as a binary indicator vector based on its tissue origin, resulting in binary distance matrix (0 for the same tissue origin, 1 otherwise). To evaluate the effect of mutation status, copy number and gene expression profile on congruence, we made three combinations of the data sets. The first set contains only the mutation status. The second set contains mutation and copy number, and in the final set, all the three data sets were combined. We computed GFA using all three combinations, and used the factors for common components shared by each data set in the congruence analysis.

### External data resources

We selected the genes that were common in GFA analyses between TAS and each one of the genomic and molecular profiling data sets (gene expression, copy number and mutation status). We investigated the cancer relevance of the selected genes using QIAGEN's IngenuityPathway Analysis (IPA; QIAGEN Redwood City, www.qiagen.com/ingenuity). We retrieved mutation information of the selected genes in various cancer patients from The Cancer Genome Atlas (TCGA) resource. The TCGA data were downloaded and extracted using the ‘cgdsr’ R package from cBioPortal (Cerami et al., 2012; Gao et al., 2013). The overall mutational frequency across all the cancers in the TCGA panel as well as the frequency in each cancer type was calculated.

### Cluster robustness analysis

To assess how strongly the observed data support the clustering solution, we used the ‘pvclust’ R package (Suzuki and Shimodaira, 2006), which computes multiscale bootstrap analysis to assess robustness of the detected clusters. The robustness value ranges between 0% (not robust) to 100% (highly robust). We used the cosangle distance method and Ward's linkage algorithm to cluster the cell lines based on their drug response profiles, such as DSS, AUC, pIC_50_ and the corresponding target addiction profiles. The data were permuted 10,000 times for assessment of the cluster robustness. We defined a linear weight between 0.1 and 1 that is proportional to the height of the sub-cluster in the dendrogram. The obtained robustness value of each sub-cluster was multiplied by its respective linear weight to obtain a height-normalized robustness value.

### Addition of extra cell lines

Beyond the 107 cell lines in our original analysis, we later selected 20 additional cell lines, which were tested with 134-137 compounds. We added the TAS profiles of the 20 new cell lines to the original clustering based on the TAS profile of the 107 cell lines, and used the same distance function and clustering algorithm to re-compute new clusters. The complete panel of cell lines was divided into eight sub-clusters (supplementary material Fig. S7). Within each sub-cluster, we performed Spearman correlation analysis between the newly added cell lines and the original cell lines within the sub-cluster to evaluate their average similarity in gene expression and copy number. To assess the statistical significance of the correlation coefficients, we computed the background null distribution as pairwise Spearman correlation coefficients of every pair of the cell lines.

### Application to leukemia patients

For the leukemia case study, we extracted the drug screening and clinical data for 151 primary leukemia patient samples (Tyner et al., 2013). In this study, the drug sensitivity profiling was carried out using 66 kinase inhibitors. Each compound's sensitivity was screened across four serial dilutions of the compound. The cell viability at each dilution was measured and normalized with no drug viability. We used the available viability data from the study for logistic curve fitting and calculated the DSS. We made use of the compound-target interactions reported in the original study by Tyner et al. (2013), which were based on the large-scale kinase inhibitor selectivity assay of Davis et al. (2011). The study by Tyner et al. (2013) also provides clinical information for some of the patients, including diagnosis and demographics, white blood cell count, karyotypes, cytogenetics and the mutational status of a selected set of key leukemia genes, such as *FLT3-*ITD.

## Notes

This article is part of a subject collection on Model Systems in Drug Discovery: from Bench to Patient. See related articles in this collection at http://dmm.biologists.org/cgi/collection/drugdiscovery
